# Attenuation of renal fibrosis after unilateral ureteral obstruction in mice lacking the N-type calcium channel

**DOI:** 10.1371/journal.pone.0223496

**Published:** 2019-10-09

**Authors:** Keiichiro Mishima, Masao Nakasatomi, Shunsuke Takahashi, Hidekazu Ikeuchi, Toru Sakairi, Yoriaki Kaneko, Keiju Hiromura, Yoshihisa Nojima, Akito Maeshima

**Affiliations:** Department of Nephrology and Rheumatology, Gunma University Graduate School of Medicine, Maebashi, Japan; Kawasaki Ika Daigaku, JAPAN

## Abstract

The N-type Ca^2+^ channel (Cav2.2) is distributed in sympathetic nerves that innervate the tubules, the vessels, and the juxtaglomerular granular cells of the kidney. However, the role of N-type Ca^2+^ channels in renal disease remains unknown. To address this issue, Cav2.2 knockout mice were utilized. Immunoreactive Cav2.2 was undetectable in normal kidneys of C57BL/6N mice, but it became positive in the interstitial S100-positive nerve fibers after unilateral ureteral obstruction (UUO). There were no significant differences in mean blood pressure, heart rate, and renal function between wild-type littermates and Cav2.2-knockout mice at baseline, as well as after UUO. Cav2.2 deficiency significantly reduced the EVG-positive fibrotic area, alpha-SMA expression, the production of type I collagen, and the hypoxic area in the obstructed kidneys. The expression of tyrosine hydroxylase, a marker for sympathetic neurons, was significantly increased in the obstructed kidneys of wild-type mice, but not in Cav2.2-knockout mice. These data suggest that increased Cav2.2 is implicated in renal nerve activation leading to the progression of renal fibrosis. Blockade of Cav2.2 might be a novel therapeutic approach for preventing renal fibrosis.

## Introduction

Interstitial fibrosis is the hallmark of various kidney diseases regardless of its cause[1]. Causative roles for inflammation, fibroblast activation, tubular and microvascular injury, and apoptosis have been established in the development of tubulointerstitial fibrogenesis[2]. Using animal models, various molecules including cytokines, chemokines, angiogenic factors, and growth factors that contribute to the progression of renal fibrosis have been identified[3].

Renal sympathetic nerves innervate the tubules, the vessels, and the juxtaglomerular granular cells[4]. These nerves are also distributed to both afferent and efferent arterioles in the glomeruli and contribute to the regulation of renal blood flow and the glomerular filtration rate[5, 6]. Recently, overactivation of the sympathetic nervous system has been thought to contribute to renal injury, and renal sympathetic nerves are an important effector of renal damage progression in various kidney diseases. Elevated plasma norepinephrine[7] and increased sympathetic nerve activity and plasma renin activity[8] were observed in patients with chronic renal diseases. It has been recently reported that reduction of sympathetic nerve activity by renal denervation could prevent both fibrogenesis and the inflammatory cascade in a mouse unilateral ureteral obstruction (UUO) model[9]. Collectively, it is considered that overactivation of renal sympathetic nerve activity is one of the factors that accelerate renal fibrosis[10, 11].

N-type Ca^2+^ channel blockade has been shown to be effective for the treatment of several diseases. For example, the expression of N-type Ca^2+^ channels was elevated in brain regions susceptible to ischemia after ischemic injury[12]. A selective N-type Ca^2+^ channel antagonist protects against ischemic brain injury[13, 14]. In DOCA-salt hypertensive rats, myocardial fibrosis was significantly improved by cilnidipine, an N-type Ca^2+^ channel antagonist, suggesting the protective effects of N-type Ca^2+^ channel inhibition on cardiovascular remodeling[15]. Similarly, it has been demonstrated that N-type Ca^2+^ channels play a role in renal injury, and their blockade elicits renoprotection in several hypertensive rats[16–20]. In l-NAME/SHR-exacerbated nephrosclerosis model, cilnidipine could reverse the severe renal hemodynamic and glomerular dynamic changes [16]. Cilnidipine also provided superior protection against renal damage compared with amlodipine in SHR/ND model[17] as well as in Dahl S rats given an HSD[20]. Cilnidipine inhibited renal dysfunction, sympathetic nerve activity and renal renin-angiotensin-aldosterone system in the DOCA-salt group[19]. Recently, it was also shown that Cav2.2 inhibition exerts renoprotective effects against the progression of diabetic nephropathy, partly by protecting podocytes[21].

N-type Ca^2+^ channels are densely distributed in the sympathetic nervous system and regulate neurotransmitter release from the nerve endings of sympathetic neurons[22]. However, the role of N-type Ca^2+^ channel in renal fibrosis remains unclear. To clarify this issue, N-type Ca^2+^ channel-knockout mice were used in this study. The findings demonstrated that the N-type Ca^2+^ channel is increased in the interstitial nerve fibers of obstructed fibrotic kidneys. Deficiency of the N-type Ca^2+^ channel significantly attenuated the fibrotic changes of the kidneys after UUO at least partly by the reduction of renal sympathetic nerve activation. Blockade of N-type Ca^2+^ channels might represent a novel effective therapeutic strategy to prevent or limit progression of renal fibrogenesis.

## Results

### Expression of Cav2.2 in obstructed kidneys

**Fig 1:**
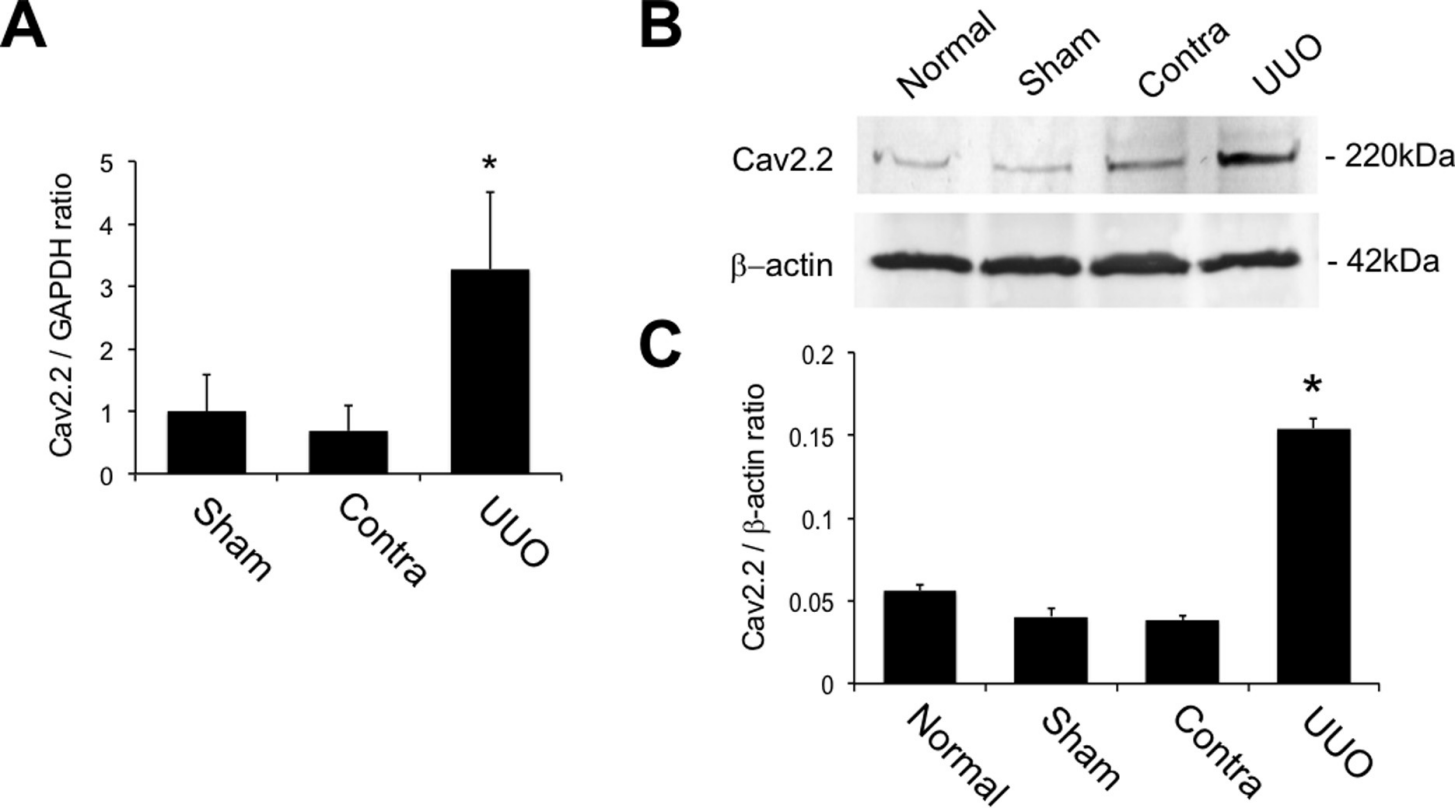
Expression and localization of Cav2.2 in obstructed kidneys. A: mRNA expression of Cav2.2 in sham-operated, contralateral, and obstructed kidneys is examined by real-time PCR. Values are means ± S.E. (n = 5). *P < 0.05 vs. sham. B: Production of Cav2.2 in normal, sham-operated, contralateral, and obstructed kidneys is examined by Western blotting. C: Quantitative analysis of Cav2.2 production. The intensity of each band is measured, and the Cav2.2/beta-actin ratio is calculated. Values are means ± S.E. (n = 5). *P < 0.05 vs. normal.

To examine the role of Cav2.2 in renal fibrosis, the mRNA expression of Cav2.2 in kidneys after UUO was first investigated by real-time PCR. The mRNA expression of Cav2.2 was significantly increased in the obstructed kidneys compared to normal or contralateral kidneys (Fig 1A). Western blot analysis demonstrated that Cav2.2 protein was slightly detected in normal, sham-operated, and contralateral kidneys, but it was abundantly present in the obstructed kidneys (Fig 1B). Quantitative analysis showed a significant increase in the production of Cav2.2 in the obstructed kidneys compared to that in normal kidneys (Fig 1C).

**Fig 2:**
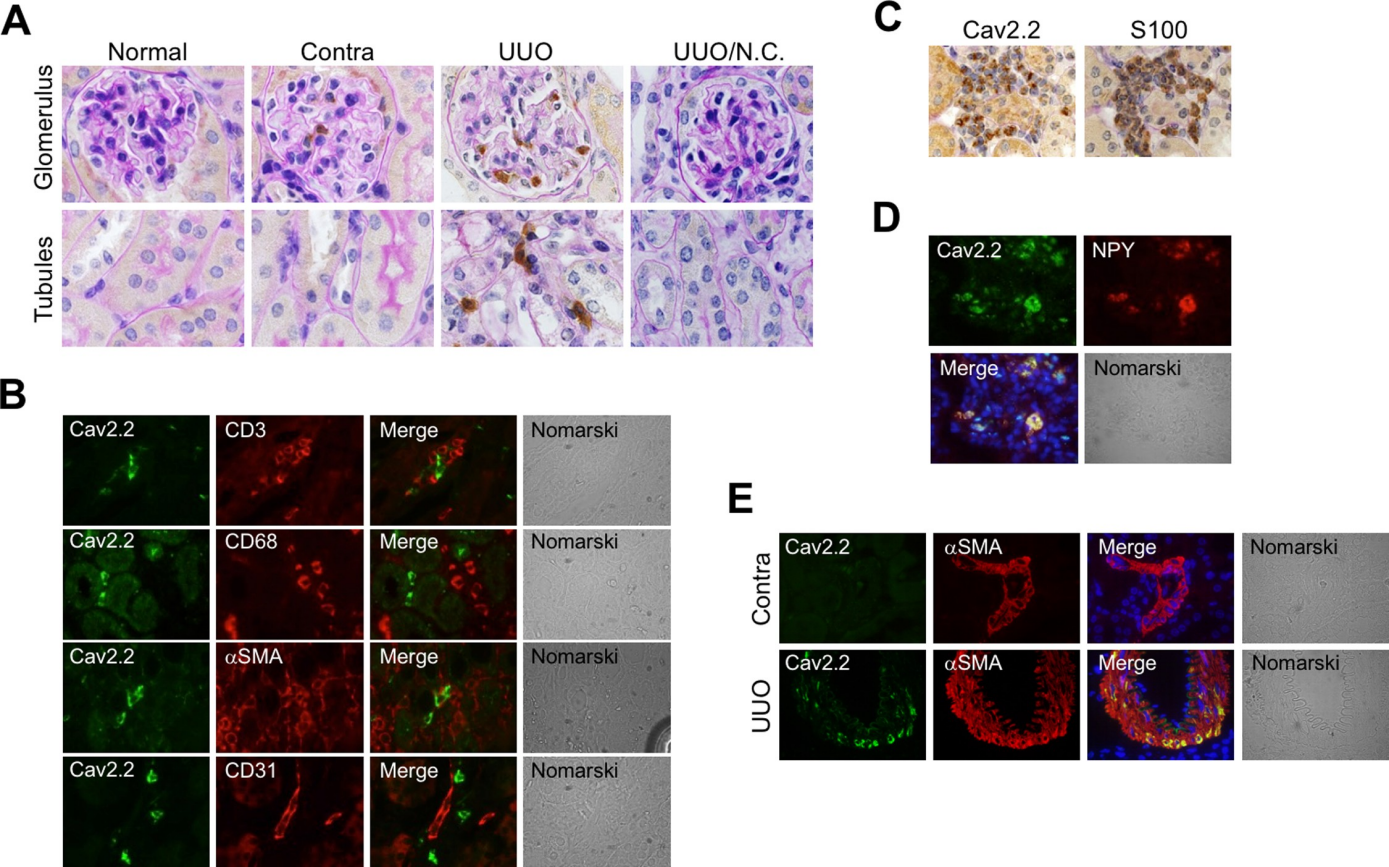
Localization of Cav2.2-positive cells in obstructed kidneys. A: Localization of Cav2.2 in normal, contralateral, and obstructed kidneys at 7 days after operation is examined by immunostaining. Sections were counterstained with PAS. Cav2.2 (brown). Magnification: ×1000. N.C., negative control. B: Double staining of Cav2.2 with CD3, CD68, alpha-SMA, or CD31 in the obstructed kidneys 7 days after operation. Cav2.2 (green), Markers (red), DAPI (blue). Magnification: ×1000. C: Immunostaining of Cav2.2 and S100 in the obstructed kidneys 7 days after operation using serial sections. Cav2.2, S100 (brown). Magnification: ×1000. D: Co-localization of Cav2.2 with neuropeptide Y in the obstructed kidneys at 7 days after operation. Cav2.2 (green), neuropeptide Y (red), DAPI (blue). Magnification: ×1000. E: Localization of Cav2.2 with alpha-SMA in contralateral and obstructed kidneys at 7 days after operation. Cav2.2 (green), alpha-SMA (red), DAPI (blue). Contra, contralateral. Magnification: ×1000.

Immunoreactive Cav2.2 was not observed in normal kidneys (Fig 2A). Cav2.2-positive cells were detected in the glomeruli of contralateral kidneys. In contrast, an intense Cav2.2 signal was present in glomeruli, as well as in the interstitum, of the obstructed kidneys (Fig 2A). The origin of Cav2.2-positive cells was further examined by double-staining of Cav2.2 with several interstitial cell markers. Cav2.2 was not co-localized with CD3-positive T-lymphocytes, CD68-positive macrophages, α-SMA-positive myofibroblasts, and CD31-positive capillary endothelial cells (Fig 2B). Cav2.2 was co-localized with interstitial nerve fibers positive for S100 (Fig 2C) or neuropeptide Y (Fig 2D). Cav2.2 was also detected in α-SMA-positive vascular smooth muscle cells of the obstructed kidneys, but not in those of contralateral kidneys (Fig 2E). These data suggest that Cav2.2 was upregulated in the interstitial nerves, as well as in vascular smooth muscle cells, during renal fibrosis.

### Physiological parameters of Cav2.2-knockout mice

**Fig 3:**
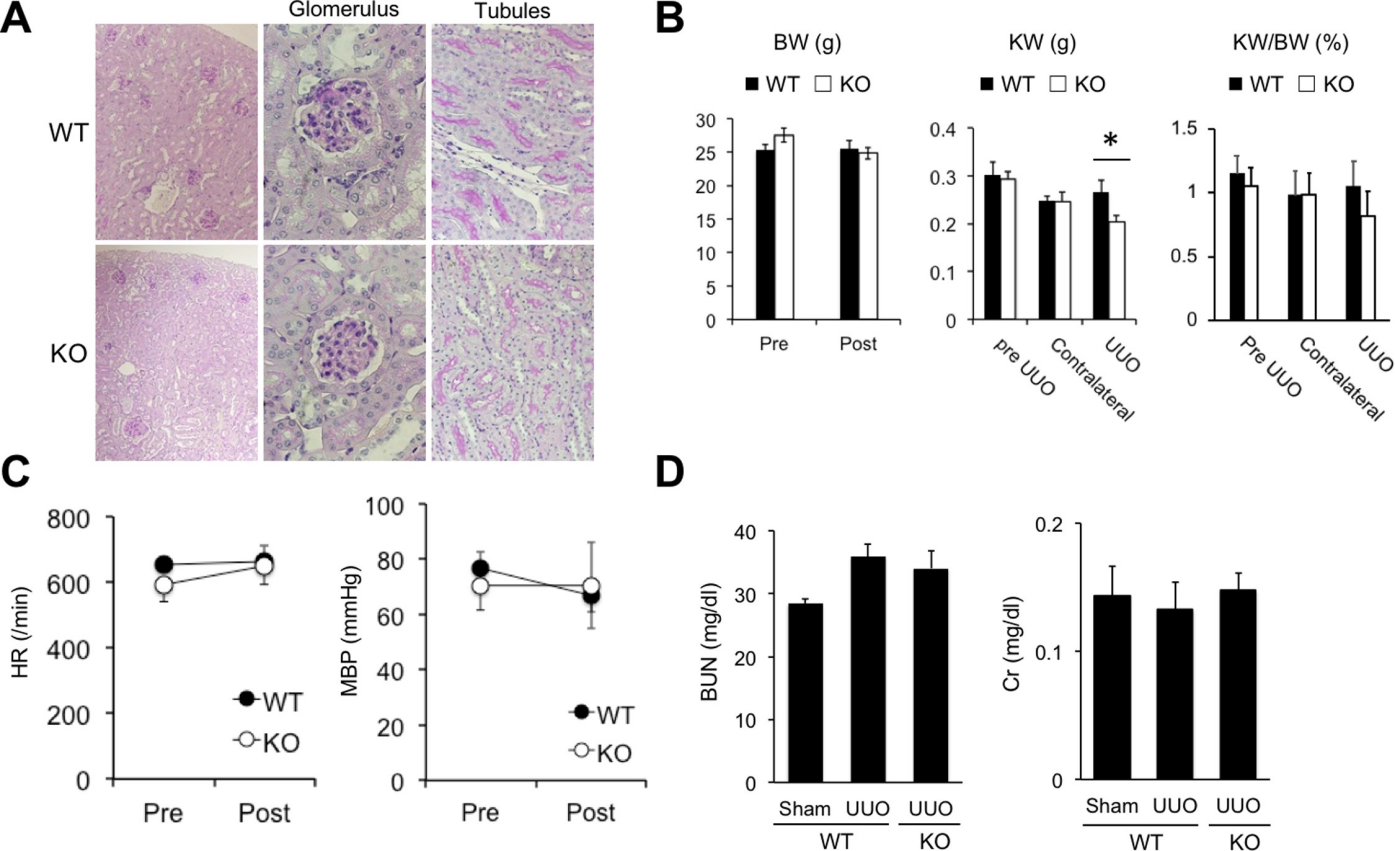
Physiological parameters of Cav2.2-knockout mice. A: PAS-stained sections of wild type and knockout mice kidneys. Magnification: ×100 (left column) and ×400 (middle and right column). B: Body weight (BW) and kidney weight (KW) of wild type and knockout mice before (Pre) and 14 days after UUO (Post). Values are means ± SE (n = 6). *P<0.05 vs. wild type mice. C: Heart rate and mean blood pressure in wild type and knockout mice before (Pre) and 14 days after UUO (Post). D: Renal function in wild type and knockout mice 14 days after UUO. Values are means ± S.E. (n = 5). BUN, blood urea nitrogen. Cr, creatinine.

To further clarify the role of Cav2.2 in renal fibrosis, Cav2.2-knockout mice were evaluated[23]. It has been reported that these knockout mice have a normal life span and are free from apparent behavioral defects. Cav2.2-knockout mice have a higher pain threshold[24–26], but they display no obvious changes in heart, kidney, lung, liver, or spleen. As reported previously[23], gross morphology and glomerular or tubular structures of Cav2.2-knockout mice kidneys were indistinguishable from those of wild type mice (Fig 3A). When UUO was induced in these knockout mice, no significant differences in body weight, kidney weight, heart rate, and mean blood pressure were found between wild type mice and Cav2.2-knockout mice before UUO (Fig 3B and 3C). The kidney weight of Cav2.2-knockout mice was significantly smaller than that of wild type mice (Fig 3B). No significant differences in the levels of blood urea nitrogen and serum creatinine were found between Cav2.2-knockout versus wild type mice before UUO as well as at 14 days after UUO (Fig 3D).

### Fibrotic changes in the obstructed kidney of Cav2.2-knockout mice

**Fig 4:**
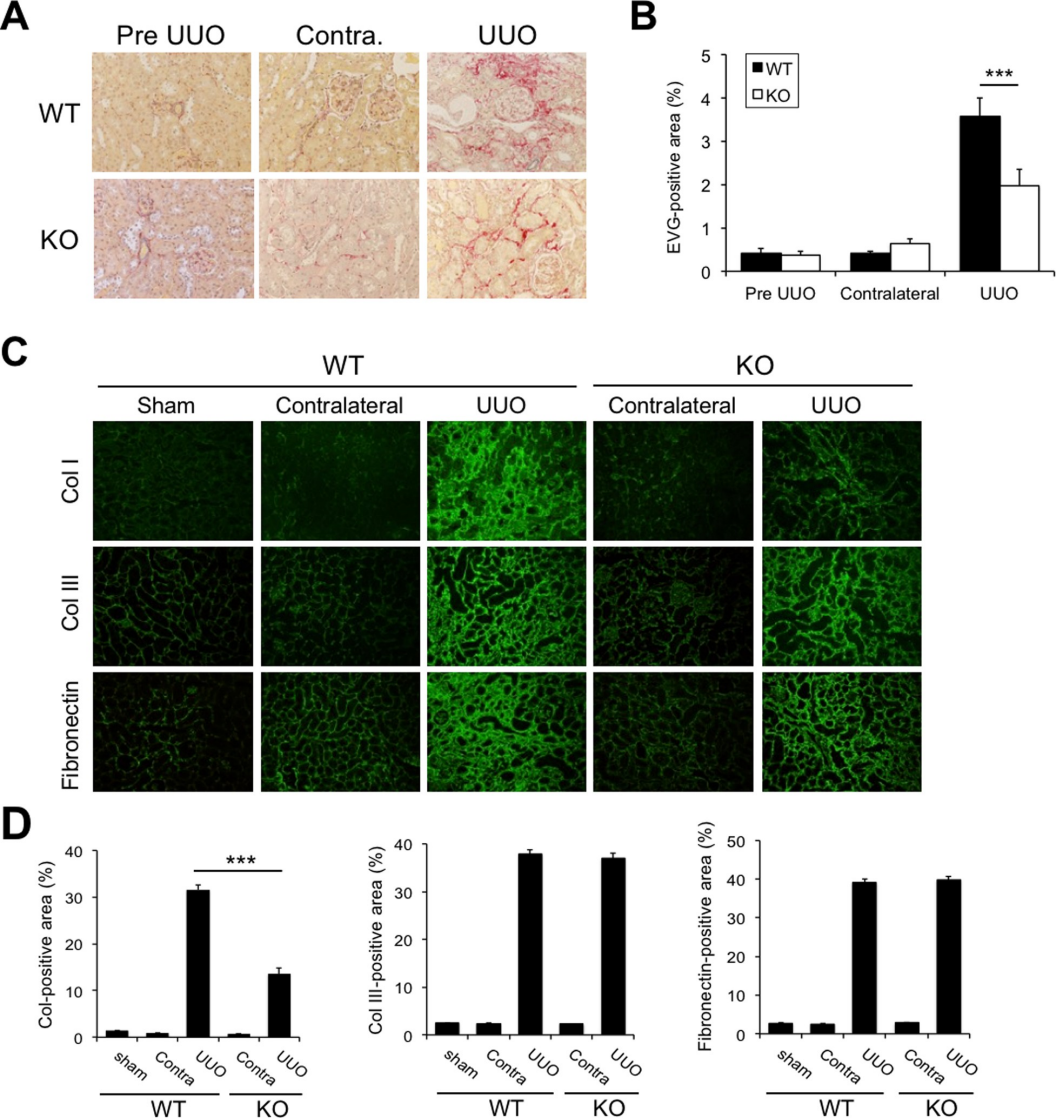
Histological changes of Cav2.2-knockout mice kidneys after UUO. A: Fibrotic changes in obstructed kidneys are assessed by EVG staining. Magnification: ×200. B: Quantitative analysis of the EVG-positive area. Values are means ± S.E. (n = 5). ***P < 0.001. C: Production of type I collagen (Col I), type III collagen (Col III), and fibronectin in sham-operated, contralateral, and obstructed kidneys 14 days after operation are examined by immunostaining. Col, Col III, fibronectin (green), DAPI (blue). Magnification: ×200. Representative images are shown. D: Quantitative analysis of extracellular matrix production. Type I collagen (D), type III collagen (E), and fibronectin (F) -positive areas in sham-operated, contralateral, and obstructed kidneys are measured. Values are means ± S.E. (n = 5). N.S., not significant. *p<0.05, **p<0.01. Magnification, ×1000.

To evaluate the fibrotic changes of the kidneys after UUO, we first used Azan staining, but there were many nonspecific signals in the apical site of renal tubular lumen. Then, we used EVG staining in the present study. Both wild type and Cav2.2-knockout mice showed renal tubulointerstitial fibrosis at 7 days after UUO (Fig 4A). Quantitative analysis demonstrated that EVG-positive fibrotic areas were significantly decreased in Cav2.2-knockout mice kidneys compared with wild type mice kidneys (Fig 4B). Unfortunately, it was difficult to find nerve fiber endings in EVG-stained section, which unable us to examine whether Cav2.2 knockout improved global renal fibrosis or the specific area only in the localized nerve fiber ending. Increased expressions of extracellular matrix components such as type I collagen, type III collagen, and fibronectin were observed in the obstructed kidney cortex of both wild type and Cav2.2-knockout mice (Fig 4C). Sham-operated and contralateral kidneys did not display significant fibrosis. Quantitative analysis showed that the type I collagen-positive area in the obstructed kidneys of Cav2.2-knockout mice was significantly decreased compared with that in wild type mice (Fig 4D). No significant difference was observed in the type III collagen-positive area and the fibronectin-positive area between wild type and Cav2.2-knockout mice (Fig 4D).

### Expressions of epithelial and mesenchymal markers in the obstructed kidneys of Cav2.2-knockout mice

**Fig 5:**
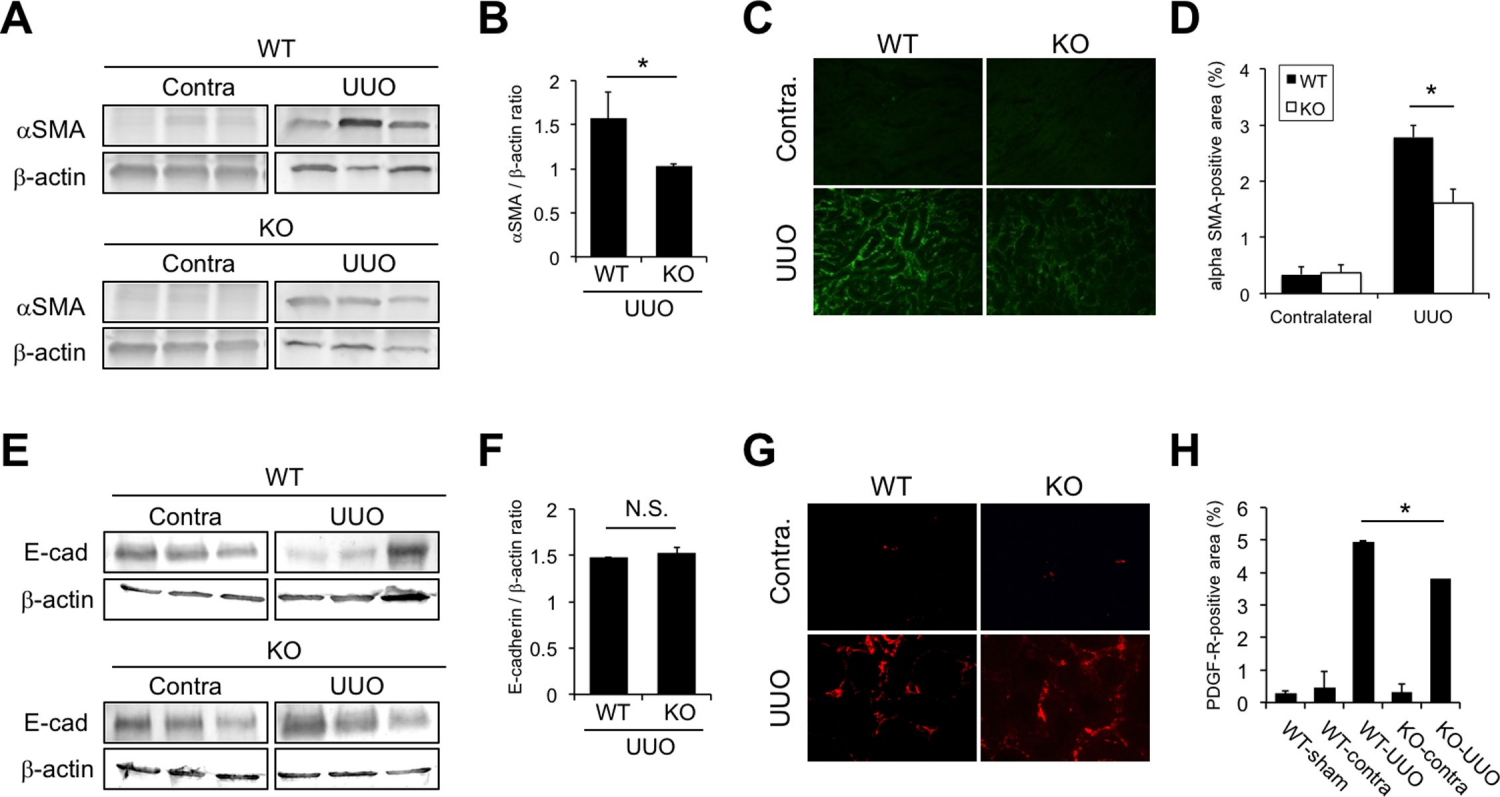
Expression of epithelial and mesenchymal markers in obstructed kidneys. A: Expression of alpha-SMA in the contralateral and obstructed kidneys 7 days after UUO is examined by Western blot analysis. B: Quantitative analysis of alpha-SMA production. Values are means ± S.E. (n = 5). *P < 0.05. C: Expression of alpha-SMA in contralateral and obstructed kidneys is examined by immunostaining. Magnification: ×200. D: Quantitative analysis of the alpha-SMA-positive area. Values are means ± S.E. (n = 5). *P < 0.05. E: Expression of E-cadherin in the contralateral and obstructed kidneys 7 days after UUO is examined by Western blot analysis. F: Quantitative analysis of E-cadherin production by Western blot analysis. Values are means ± S.E. (n = 5). N.S., not significant. G: Expression of PDGF-R-beta in contralateral and obstructed kidneys is examined by immunostaining. Magnification: ×200. H: Quantitative analysis of the PDGF-R-beta-positive area by immunostaining. Values are means ± S.E. (n = 5). *P < 0.05.

The expressions of alpha-SMA, E-cadherin, and PDGF-R-beta in the UUO kidneys were also examined by Western blot analyses. The expression of alpha-SMA, which was undetectable in contralateral kidneys, was upregulated in the obstructed kidneys of both wild type and Cav2.2-knockout mice (Fig 5A). Quantitative analysis showed a significant decrease in the expression of alpha-SMA in Cav2.2-knockout mice compared to that in wild type mice (Fig 5B). Immunostaining showed that alpha-SMA-positive cells were detected in the obstructed kidneys, but not in contralateral kidneys of both wild type and Cav2.2-knockout mice (Fig 5C). The alpha-SMA-positive area was significantly reduced in Cav2.2-knockout mice compared to that in wild type mice (Fig 5D). The expression of E-cadherin, an epithelial cell marker, was not significantly different in the obstructed kidneys between wild type and knockout mice (Fig 5E and 5F). The positive area for PDGF-R-beta, a marker for pericytes, was significantly increased after UUO, and it was significantly reduced in Cav2.2-knockout mice kidneys compared to that in wild type mice kidneys (Fig 5G and 5H).

### Hypoxia and cell proliferation in the obstructed kidneys of Cav2.2-knockout mice

**Fig 6:**
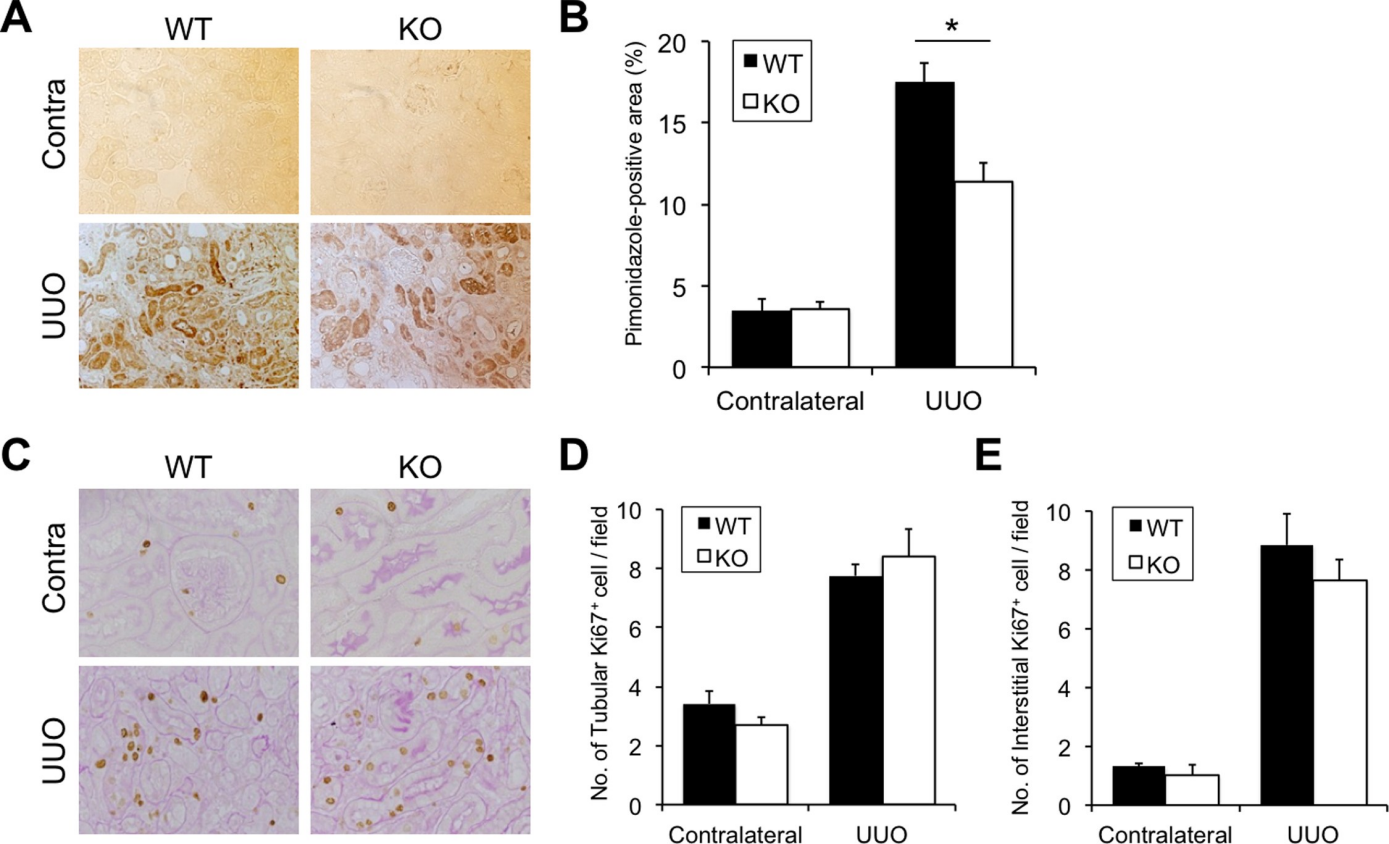
Hypoxic area and cell proliferation in obstructed kidneys. A: Detection of the hypoxic area. Localization of pimonidazole is examined by immunostaining. Magnification, ×1000. Positive area (brown). B: Quantitative analysis of the hypoxic area. **p<0.01. C: Cell proliferation is examined by Ki67 staining. Magnification, ×1000. D, E: Quantitative analysis of Ki67-positive nuclei in the tubular area (D) and the interstitial area (E). Values are means ± S.E. (n = 5). N.S., not significant.

The effect of Cav2.2 deficiency on renal hypoxia, one of the causes of renal fibrosis, in the obstructed kidneys was further investigated by pimonidazole immunostaining. The pimonidazole-positive hypoxic area, which was absent in contralateral kidneys, became detectable in the obstructed kidneys of both wild type and knockout mice (Fig 6A). The pimonidazole-positive hypoxic area was significantly reduced in knockout mice obstructed kidneys compared to that in the wild type mice obstructed kidneys (Fig 6B). Cell proliferation in the kidney after UUO was also examined by Ki67 immunostaining. Ki-67-positive nuclei were observed in the kidneys of both wild type and knockout mice after UUO (Fig 6C). There were no significant differences in the numbers of Ki67-positive tubular cells (Fig 6D) and interstitial cells (Fig 6E) in the obstructed kidneys between wild type and knockout mice.

### Expressions of tyrosine hydroxylase and dopamine hydroxylase in the obstructed kidneys of Cav2.2-knockout mice

**Fig 7:**
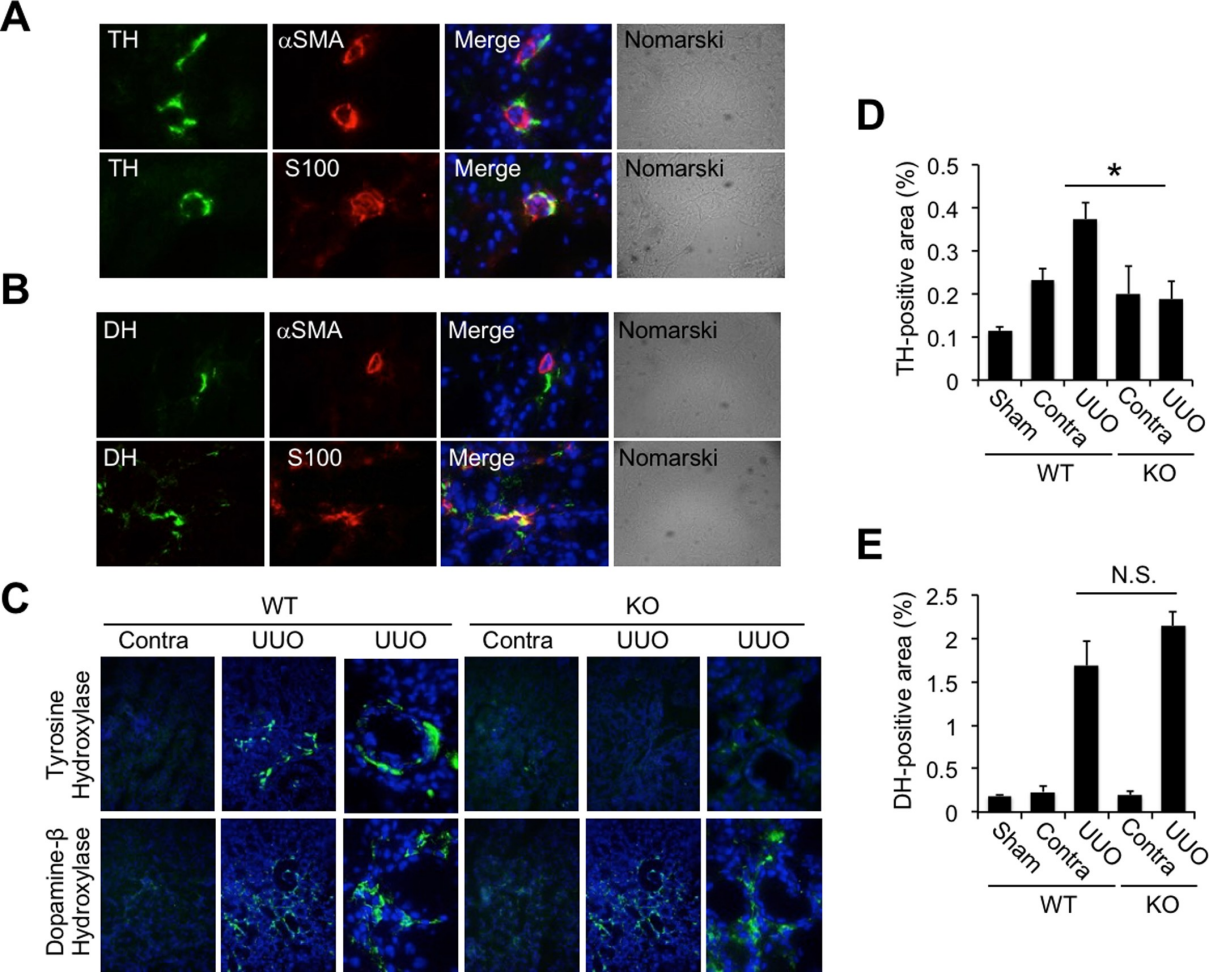
Localization of tyrosine hydroxylase and dopamine hydroxylase in obstructed kidneys. A, B: Localization of tyrosine hydroxylase (TH) (A) and dopamine beta hydroxylase (DH) (B) in normal kidneys is examined by immunostaining. TH, DH (green), alpha-SMA (red), DAPI (blue). Magnification: ×1000. C: Expressions of TH and DH in normal, sham-operated, contralateral, and obstructed kidneys are examined by immunostaining. Representative images are shown. Magnification, ×1000. TH, DH (green), DAPI (blue). D: Quantitative analysis of the TH-positive area. Values are means ± S.E. (n = 5). *p<0.05. E: Quantitative analysis of the DH-positive area. Values are means ± S.E. (n = 5).

To examine the effect of Cav2.2 blockade on renal sympathetic nerve activity, the expressions of tyrosine hydroxylase (TH) or dopamine hydroxylase (DH), markers of sympathetic nerve fibers, in the obstructed kidneys were analyzed by immunostaining. In normal kidneys, TH (Fig 7A), as well as DH (Fig 7B), was co-localized with S100-positive cells adjacent to alpha-SMA-positive cells, suggesting that both TH and DH were expressed in the peripheral nerves attached to vascular smooth muscle cells in normal mice kidneys. The expressions of TH and DH in the obstructed kidneys were then examined. Expression of TH was significantly increased in the obstructed kidneys compared to that in contralateral kidneys of wild type mice (Fig 7C). In contrast, the increase of TH expression in the kidneys after UUO was absent with Cav2.2 deficiency (Fig 7D). DH expression was also enhanced in the kidneys of wild type mice after UUO (Fig 7C). Cav2.2 deficiency did not affect the expression level of DH in the obstructed kidneys (Fig 7E).

## Discussion

We previously reported that upregulated expression of N-type Ca^2+^ channels was observed in obstructed kidneys, and blockade of N-type Ca^2+^ channels by cilnidipine significantly reduced renal fibrosis in a rat UUO model[27]. Consistent with these results, our data demonstrated that Cav2.2 activation is involved in the fibrotic process of obstructed kidneys. The expression of Cav2.2 was increased in the interstitial nerve fibers in the obstructed kidneys, but not in normal or contralateral kidneys (Figs 1 and 2). Cav2.2-knockout mice have no abmormalities in physiological parameters such as heart rate or mean blood pressure (Fig 3), suggesting that other Ca^2+^ channels could compensate for the functions of mutated Cav2.2 channels. Nevertheless, Cav2.2 deficiency significantly reduced the EVG-positive fibrotic area, alpha-SMA expression, the production of type I collagen, and the hypoxic area in the obstructed kidneys (Figs 4–6). Although the mechanism by which Cav2.2 was increased in the interstitial nerve fibers during renal fibrosis remains unknown, it is quite likely that Cav2.2 activation contributes to the progression of renal fibrosis. Cav2.2 would be a novel therapeutic target to prevent this process.

Both TH and DH are catalytic enzymes necessary for the biosynthesis of catecholamines in sympathetic nerve fibers. It has been reported that TH immunoreactivity is associated with norepinephrine content within nerve terminals[28], indicating that TH is a valuable indicator reflecting local sympathetic nerve activity. In the present study, TH and DH were localized in the perivascular S100-positive nerve fibers, and both expression levels were significantly upregulated in the obstructed kidneys compared to in the contralateral kidneys (Fig 7), suggesting the activation of renal sympathetic nerve activity in this UUO model. Given that increased expression of TH was not observed in the obstructed kidneys of Cav2.2-knockout mice (Fig 7), it is possible that Cav2.2 blockade reduced overactivation of the renal sympathetic nervous system in the obstructed kidneys.

**Fig 8:**
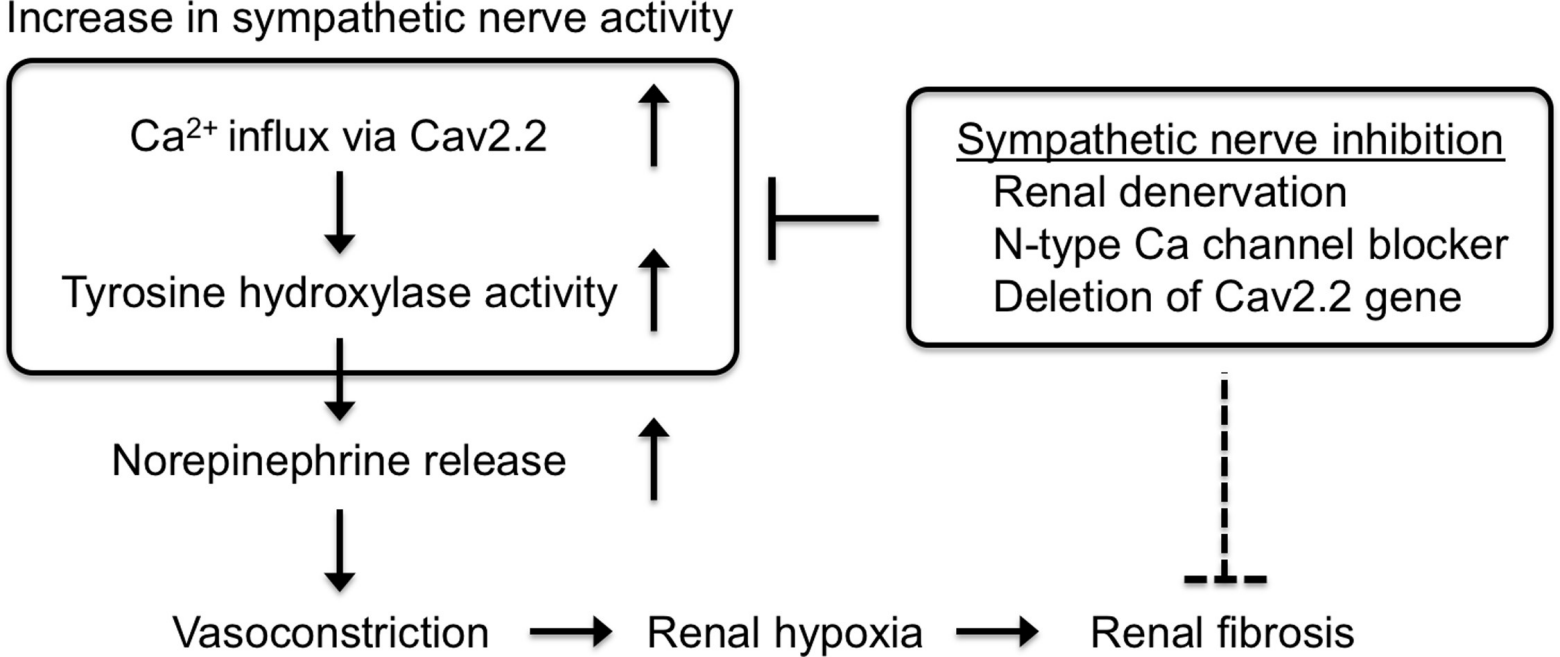
Possible mechanism how sympathetic nerve inhibition improves renal hyopxia leading to the reduction of renal fibrosis.

Voltage-dependent Ca^2+^ channels mediate Ca^2+^ entry into cells and play key roles in muscle contraction, neuronal excitability control, and the release of neurotransmitters. In addition, Ca^2+^ channel-mediated Ca^2+^ entry is also involved in transcriptional regulation of various gene expressions. Previous data suggest possible associations of the N-type Ca^2+^ channel with TH expression and norepinephrine release. It has been reported that increased Ca^2+^ influx following depolarization induces the activation of TH, leading to an increase in the rate of norepinephrine synthesis in sympathetic neurons[29]. In contrast, N-type Ca^2+^ channel blockade completely abolished both mRNA and protein induction of TH after electrical stimulation in primary sensory neurons[30]. Sympathetic N-type Ca channel inhibition also reduces norepinephrine release during colitis[31]. Consistent with this, in the present study, expressions of both Cav2.2 and TH were increased in the obstructed kidneys, and Cav2.2 blockade significantly reduced TH expression (Fig 7). These data suggest that Ca^2+^ entry via Cav2.2 is a trigger of TH gene transcription in the interstitial nerve fibers during renal fibrosis. It was also observed that the pimonidazole-positive hypoxic area in the obstructed kidneys was significantly increased compared to that in the contralateral kidneys (Fig 6). In addition, the hypoxic area was significantly smaller in the obstructed kidneys of Cav2.2-knockout mice than of wild type mice (Fig 6). Norepinephrine induces vasoconstriction via adrenogenic receptors expressed in vascular smooth muscle cells, leading to hypoxia in adjacent tissue. Norepinephrine also induces the release of profibrotic factors such as TGF-beta1 and CTGF in cultured renal tubular cells[9]. Taken together, Cav2.2 blockade might prevent renal fibrosis by inhibiting TH-mediated norepinephrine production in the obstructed kidneys (Fig 8). Further study will be necessary to clarify the role of Cav2.2 in the progression of renal fibrosis in humans.

## Materials and methods

### Reagents

Antibodies used in this study were as follows: goat anti-mouse CD3-epsilon antibody, goat anti-CD31 antibody, rabbit anti-TH antibody, goat anti-NPY antibody, rabbit polyclonal anti-fibronectin antibody (Santa Cruz Biotechnology, Santa Cruz, CA), mouse monoclonal anti-alpha-smooth muscle actin (SMA) (Sigma, St. Louis, MO), rabbit anti-Cav2.2 (Alomone Labs, Jerusalem, Israel), mouse anti-beta-actin antibody (BioVision Research Products, CA), goat anti-E-cadherin antibody (BD Transduction Laboratories, Franklin Lakes, NJ), goat polyclonal anti-collagen I, goat polyclonal anti-collagen III (Southern Tech, Birmingham, AL), rabbit anti-DH antibody, mouse anti-CD68 antibody (Abcam, Cambridge, UK), rabbit anti-Ki67 antibody, and mouse anti-S100 antibody (Thermo Scientific Japan, Tokyo, Japan).

### Mice

Generation and phenotype characterization of mice with a nonfunctional alpha1B subunit of VDCCs (Cav2.2^-/-^ knockout mice) (provided by Dr. Miyamoto, Eisai Co., Ltd., Tsukuba, Japan) have been previously described[23]. Both Cav2.2^+/+^ wild-type littermates and Cav2.2^-/-^ knockout mice were offspring of heterozygous (+/−) mice with the C57BL/6 genetic background. For the present experiments, 16-week-old male Cav2.2^-/-^ knockout mice (n = 6), Cav2.2^+/+^ wild-type mice (n = 6), and C57BL/6 mice (n = 6) (Japan SLC, Inc., Hamamatsu, Japan) were housed under specific pathogen-free conditions and provided with autoclaved food and sterile water *ad libitum*. Mice were randomly tested and documented to be serologically negative for common murine pathogens. All animal experiments were performed in accordance with Fundamental Guidelines for Proper Conduct of Animal Experiment and Related Activities in Academic Research Institutions and were approved by the Ethics Review Committee for Animal Experimentation of Gunma University (Approved number: 12–054).

### Unilateral ureteral obstruction (UUO)

UUO was performed as described previously[32]. Briefly, after induction of general anesthesia by intraperitoneal injection of pentobarbital (50 mg/kg body weight), the abdominal cavity was exposed via a midline incision, and the left ureter was ligated at two points with 4–0 silk. At the indicated times after UUO, mice were sacrificed, and the kidneys were removed for histological examination. Ureteral obstruction was confirmed by observing dilation of the pelvis and proximal ureter and collapse of the distal ureter. Sham-operated mice had their ureters exposed and manipulated but not ligated. Arterial blood pressure was measured at the indicated time points using a programmable apparatus by the tail cuff method. All mice were euthanized by cervical dislocation while under general anesthesia. Serum creatinine and blood urea nitrogen (BUN) levels were assessed by a Hitachi 7180 autoanalyzer (Hitachi High-Technologies, Tokyo, Japan).

### Real-time PCR

Tissues were homogenized using a micro-homogenator, and total RNAs were extracted using RNAiso (Takara, Tokyo, Japan). First-strand cDNA was made from total RNA using Super Script III First-Strand (Invitrogen, Carlsbad, CA, USA) according to the manufacturer’s instructions. Real-time PCR was performed as described previously [32] by the ABI 7300 Real-time PCR System (Applied Biosystems, Foster City, CA, USA). Reactions included 5 ul of a SYBR® Green RealtimePCR MasterMix (TOYOBO, Osaka, Japan), 0.2 ul 3′ primer, 0.2 ul 5′ primer, and 1 ul cDNA. Samples were incubated at 50°C for 2 min, then at 95°C for 5 min, followed by 40 cycles of 15 s at 95°C, and 60 s at 55°C. Mouse primers used in this study were as follows: Cav2.2 (sense and antisense: 5′-TGCCAACATCTCCATTGCT-3′ and 5′-AGTTCTGCTGCGGTGAGTTT-3′, respectively) and GAPDH (sense and antisense: 5′-TGCCAACATCTCCATTGCT-3′ and 5′-AGTTCTGCTGCGGTGAGTTT-3′). All data are expressed as the relative differences among normal, sham-operated, and contralateral or obstructed kidneys after normalization to GAPDH expression. Gels of the PCR products after quantification of the indicated genes by real-time PCR showed single bands in which the band size was the same as expected.

### Immunohistochemical analysis

Immunostaining was performed using the VECTASTAIN ABC-kit (Vector Laboratories, Burlingame, CA, USA) as described previously[33]. Briefly, paraffin sections (4-μm-thick) were deparaffinized, hydrated according to standard methods, soaked in blocking serum, and incubated with primary antibody overnight at 4°C. After washing with phosphate-buffered saline (PBS), sections were incubated with peroxidase-conjugated secondary antibody, followed by reaction with diaminobenzidine and counterstained with PAS.

Indirect fluorescent immunostaining was performed as described previously[34]. Briefly, sections were incubated with primary antibodies overnight at 4°C. After washing with PBS, sections were incubated with fluorescence-labeled secondary antibodies and 4’,6-diamidino-2’-phenylindole dihydrochloride (DAPI). Fluorescent images were recorded as described previously[32].

### Histological examination

Sections (4-um-thick) were stained with periodic acid schiff (PAS), hemaoxylin-eosin (HE), Azan and elastica-van Gieson (EVG) stains. Using EVG-stained sections, the collagenous, fibrotic areas were quantitated in 10 random cortical fields at 200x magnification using Image J software. All glomeruli and vessels were subtracted from a given field, yielding a target area of tubulointerstitium.

### Western blot analyses

Western blot analysis was performed as described previously[32]. Tissues were lysed in M-PER Mammalian Protein Extraction Reagent (PIERCE Co. Ltd, Rockford, IL). After centrifugation, supernatant was collected, and the protein concentration was determined with the BCA protein assay kit (Pierce Co. Ltd). Twenty micrograms of protein from each sample were separated by SDS-PAGE and transferred to a polyvinylidene difluoride (PVDF) membrane (Millipore, Bedford, MA). To reduce nonspecific antibody binding, the membrane was blocked with 5% nonfat milk, incubated with primary antibody for 2 hours, and washed with Tris-buffered saline (20 mM Tris-HCl, 150 mM NaCl, and 0.1% Tween 20). After incubation with alkaline phosphatase-conjugated secondary antibody for 2 hours, the membrane was incubated in BCIP/NBT solution for 10 minutes (Sigma Aldrich, St. Louis, MO). The intensity of each band was measured using Image J software.

### Cell proliferation

Quantitative analysis of Ki67-positive cells was performed by counting Ki67-positive nuclei in the tubular area or in the interstitial area from five randomly selected fields under a light microscope at ×200 magnification. The average of five determinations was calculated, and the number of tubular or interstitial Ki67-positive cells per field was quantified.

### Detection of hypoxic area

Renal tissue hypoxia was detected using pimonidazole (Hypoxyprobe™-1; Natural Pharmacia International, Inc., Belmont, MA, USA) following the manufacturer’s instructions. Briefly, pimonidazole hydrochloride (60 mg/kg body weight) was injected into mice 1 hour prior to sacrifice, and paraffin-embedded sections of the kidneys were prepared. Immunostaining using anti-pimonidazole antibody was performed as described above. The percentage of the positive area was measured using Image J software.

### Statistical analysis

The differences between means were compared by Student’s *t-*test, with *P* values of <0.05 considered significant.
